# The Hook Worm

**Published:** 1911-12

**Authors:** 


### EDITORIAL DEPARTMENT

Dr. D. R. Fly, of Amarillo, Texas, the beloved President of the State Medical Association of Texas, died of tuberculosis at Fort Worth, Texas, November 29th (ult.). Biography will appear in next issue; too late for December number.

---

The Hook Worm.—Dr. Stiles, the hook worm apostle, representing the Marine Hospital Service, has recently made a visit to Texas, lecturing in some of the larger cities on the hook worm and illustrating his lectures by stereopticon slides made from actual photographs. It will be remembered that some time ago when the subject came up a proposition was made that Texas should participate in the fund provided by Mr. Rockefeller for the study and eradication of the hook worm. The proposal was necessarily "turned down" for the reason that one of the conditions was that Texas should furnish a stipulated sum of money to be used jointly with the Rockefeller fund, and Texas had no money available for that purpose. At his late visit to Austin, November 16, I believe the proposition was not again submitted; at least no agreement was made, if, indeed, anything was said of it.

Dr. Stiles is an enthusiast on the subject and some think for that reason he rather exaggerates the effects of this little invader. Like the unknown thief in the community, everything wicked that is done is laid to him; or like the liver,—every symptom of ailment otherwise not accounted for is charged to "torpid liver." Dr. Stiles says hook worm is the cause of many cases attributed to malaria; of much indigestion; of heart disease; of much "biliousness"; of disposition to consumption; of ulcers and skin diseases, and that the mode of fatal termination is exhaustion, the result of pernicious and inveterate anemia.

His lectures are highly interesting. He is full of the subject, is a pleasant speaker, and has a store of statistics to support his contention. He declares that the surface privy is the curse of the South,—the hotbed of the hook worm; and that in the country at the schoolhouses and even at the churches there are no privies at all; but that congregation and pupils squat on the ground wherever they can get out of sight; and that in consequence of this great neglect of common decency the seep wells, springs and streams are polluted, and he had statistics to show that Americans devour—consume—imbibe—more human excrement than any people on earth,—more, he said—ten times more than the Germans, four times more than the Swiss. The insanitary conditions of the farms (premises) was his special theme. One is inclined to believe that it is a pure assumption that the Americans "are the dirtiest people in the world"—not personal uncleanliness,—though he might have included the bulk of population, people who never, or seldom, take a bath, because it must be evident that Dr. Stiles' observations did not extend to Naples, Cologne, Constantinople, Russia and China, or Thibet, Hindustan, India, Kamchatka, Lapland, the Eskimos or South Sea Islanders, Bagdad or Jerusalem.

The hook worm gains entrance to the human system through the feet, and it is they that cause "toe itch," "ground itch," "dew-itch"—so well known to all barefoot children (and, in the "tackey" country—adults as well). They make their way into the venous stream, reach the heart and lungs, thence into the stomach and intestines, where they raise their Ebenezer, locate, establish their home and propagate their kind. They bury their head in the mucous membrane of the intestines and *suck;* they live on the blood. A pair of hook worms, male and female, will produce thousands of (fertilized) eggs. The eggs being aerobic, can not hatch in the intestines, but are voided. They hatch on the ground and lie in wait for the barefooted and get in their work via the toe route, and thus is the endless chain. They get into the water as well and reach the intestines.

The remedy, Dr. Stiles says, is *thymol* and epsom salts,—dose for an adult is 45 grains, followed by salts, and repeated if necessary. He said nothing of iron, arsenic or other tonics in the resulting anemia or of air, sunshine or diet as factors in the treatment, nevertheless, the doctor is doing good missionary work.

It is a mistake to suppose that this dreadful scourge (?) is confined to the poor, ignorant and insanitary. It has been found in families of the best and cleanest,—like pellagra; pellagra invades the homes of the best, sometimes.

The profession of Texas are not a unit as to Dr. Stiles' doctrines; there is considerable opposition. In South Carolina the doctors have openly rebelled against the M. H. S. and Rockefeller interference.

